# Preparation and Microwave Absorption Properties of C@Fe_3_O_4_ Magnetic Composite Microspheres

**DOI:** 10.3390/ma12152404

**Published:** 2019-07-28

**Authors:** Youqiang Shi, Yanan Yin, Yi Zhang, Yue Hu, Weifeng Liu

**Affiliations:** 1 Aviation Key Laboratory of Science and Technology on Stealth Materials, Beijing Institute of Aeronautical Materials, Beijing 100095, China; 2 Capital Aerospace Machinery Corporation Limited, Beijing 100076, China

**Keywords:** magnetic microspheres, microwave absorption, core/shell structure, dispersion polymerization, solvothermal method

## Abstract

In this work, C@Fe_3_O_4_ magnetic microspheres were designed and prepared by a novel strategy, and the microwave absorption properties of the materials were investigated. Four kinds of monodisperse P(MAA/St) microspheres with different carboxyl content were synthesized via facile dispersion polymerization. The Fe_3_O_4_ nanoparticles were grown on the surface of P(MAA/St) to obtain P(MAA/St)@Fe_3_O_4_ microspheres. Using P(MAA/St)@Fe_3_O_4_ as the precursors, after vacuum carbonization, C@Fe_3_O_4_ were obtained. It was observed that the carboxyl content on the microspheres’ surface increased with the increasing of MAA, which made the magnetic content and maximum specific saturation magnetization of P(MAA/St)@Fe_3_O_4_ and C@Fe_3_O_4_ increase. The obtained four kinds of C@Fe_3_O_4_ microspheres had a particle size range of 4–6 μm. The microwave absorption properties indicated that the magnetic content made a difference to the microwave absorption properties of C@Fe_3_O_4_ magnetic microspheres. The microwave absorption properties of materials were determined by controlling dielectric loss, magnetic loss and impedance matching. C@Fe_3_O_4_ microspheres exhibited excellent microwave absorption properties. The maximum reflection loss could reach −45.6 dB at 12.8 GHz with 3 mm in thickness. The effective bandwidth was 5.9 GHz with RL < −10 dB. Therefore, C@Fe_3_O_4_ microspheres were lightweight and efficient microwave absorption materials.

## 1. Introduction

Electromagnetic waves have been widely used in various fields of civil and military applications, such as communications, catering, medical care, stealth, navigation, electromagnetic interference, etc. While bringing convenience to people’s daily lives and providing security for national defense, the deterioration of the electromagnetic environment caused by electromagnetic leakage cannot be ignored. Microwave absorbers have been considered as the most effective way to solve the electromagnetic leakage problem [1,2,3,4,5,6,7]. Among all the microwave absorption materials developed currently, carbon materials [8,9], magnetic materials [10,11] and their composites [12,13] are mainly used for three mechanisms of absorption and loss microwaves by absorbing materials, resistive loss, dielectric loss and magnetic loss mechanisms, respectively.

Composite particles containing magnetic materials and carbon materials are highly efficient absorbing materials, which combine the characteristics and advantages of both materials. Magnetic carbon particles with various morphologies, particle size scales, pore properties, magnetic contents, and magnetic responsiveness were developed and used as absorbing agents [14,15,16,17,18]. There are various techniques to prepare magnetic carbon composite particles, such as (1) Preparation of magnetic nanoparticles via loading or in situ polymerization in porous carbon materials [19,20]; (2) preparation of polymer-magnetic nanoparticle composites, followed by carbonization under inert conditions [21,22]; and (3) direct conversion precursor polymers with iron, cobalt and nickel [23,24]. In recent years, the research of magnetic carbon composite particles as absorbing agents has mainly focused on designing novel, reasonable structures and developing new composite methods.

Herein, C@Fe_3_O_4_ magnetic composite microspheres were prepared by the solvothermal process. The absorbing properties of the microspheres were studied and the absorbing mechanism was clarified. The effect of the surface morphology and content of Fe_3_O_4_ were investigated. The surface carboxyl group content of P(MAA/St) microspheres and C@Fe_3_O_4_ magnetic composite microspheres were regarded as promising materials in civil and defense fields with excellent absorption properties.

## 2. Materials and Methods

### 2.1. Materials

Styrene (St), α-methacrylic acid (MAA), azobisisobutyronitrile (AIBN), sodium citrate, sodium acetate, ferric chloride, ethylene glycol and anhydrous ethanol were purchased from Sinopharm Chemical Reagent Co., Ltd (Being, China). Triton-100 (TX-100) was obtained from BASF (Ludwigshafen, Germany); polyvinylpyrrolidone (PVP) was bought from Shanghai Yiyan Biotechnology Co., Ltd (Shanghai, China). All chemicals were used as received. Deionized water was taken by the AJY-2002-U ultrapure water mechanism (Chongqing Yiyang Enterprise Development Co., Ltd., Chongqing, China) throughout the whole process.

### 2.2. Preparation of Monodisperse P(MAA/St) Microspheres

The P(MAA/St) microspheres were prepared by the following procedures. Azobisisobutyronitrile (0.25 g) was weighted and dissolved by a mixture of the α-methacrylic acid and the styrene monomer. Polyvinylpyrrolidone (4.0 g) and TX-100 (0.35 g) were dissolved evenly in anhydrous ethanol. Then, the two kinds of solutions were mixed in a three-necked flask by mechanical stirring. The mixture was heated to 70 °C and held at this temperature for 7 h. Next, the obtained products were centrifuged and washed 3 times with anhydrous ethanol and dried to obtain monodisperse P(MAA/St) polymer microspheres (Table 1).

### 2.3. Preparation of P(MAA/St)@Fe_3_O_4_ Magnetic Composite Microspheres

The following shows the process of Fe_3_O_4_ particles growth on the P(MAA/St) microspheres surface by solvothermal method. Sodium citrate (0.25 g), sodium acetate (1.60 g), ferric chloride (1.00 g) and P(MAA/St) microspheres (0.7 g) were dispersed and dissolved in ethylene glycol (33 mL) by ultrasonic for 30 min. Then, the mixture was transferred to a Teflon-lined stainless steel reaction vessel and reacted at 200 °C for 10 h. After cooling to room temperature naturally, the products were washed 3 times with anhydrous ethanol and deionized water, respectively. After drying, the P(MAA/St)@Fe_3_O_4_ magnetic composite microspheres were obtained.

### 2.4. Preparation of C@Fe_3_O_4_ Magnetic Composite Microspheres

The as-prepared P(MAA/St)@Fe_3_O_4_ magnetic composite microspheres were placed in a quartz crucible and calcined in a vacuum at 550 °C for 8 h with a heating rate of 5 °C/min. Then, the C@Fe_3_O_4_ magnetic composite microspheres were obtained after cooling to room temperature with the furnace.

### 2.5. Characterization

The crystal structure of the samples was examined by X-ray diffraction (XRD) using the XRD-7000 diffractometer instrument (Shimadzu, Kyoto, Japan) (Cu target Kα radiation, diffraction beam graphite crystal monochromator, tube pressure 40 KV, tube flow 40 mA). The morphology of the materials was characterized by scanning electron microscopy (SEM) (AMERY-1000B, AMERY, AMERY, WI, USA. A vibrating-sample magnetometer (VSM, LakeShore7307, LakeShore, Columbus, OH, USA) was used to measure the magnetic properties. Heat resistance and magnetic contents of the materials were obtained by a thermal gravimetric analyzer (TGA) (HCT-1, METTLER, Zurich, Switzerland). The microwave absorption properties were evaluated using a vector network analyzer (VNA) (N5227, Agilent Technologies, Santa Clara, CA, USA).

The carboxyl contents were determined by acid-base titration. P(MAA/St) (30 mg) microspheres were added into the calibrated NaOH solution (50 mL). After magnetic stirring for 12 h, the filtrate was obtained using a microporous membrane. With methyl orange and methylene blue mixed solution as the indicator, the filtrate (15 mL) was titrated with calibrated hydrochloric acid solution three times. The surface carboxyl content of P(MAA/St) microspheres (mmoL·g^−1^) was calculated based on the equation: carboxyl group content = (N_1_V_1_ − N_2_V_2_ × 50/10)/W, where N_1_ and V_1_ were the concentration and volume of the standard sodium hydroxide solution. N_2_ and V_2_ were the concentrations of the standard hydrochloric acid solution and the volume consumed. W was the mass of the P(MAA/St) microspheres.

## 3. Results and Discussion

### 3.1. Morphology and Composition Analysis of the Synthesized P(MAA/St) Microspheres

**Figure 1:**
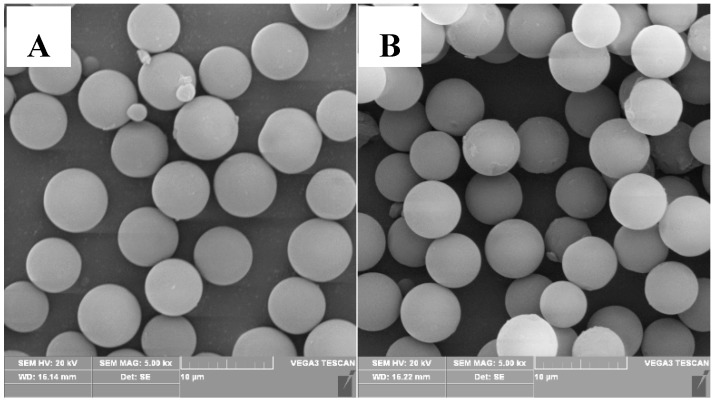

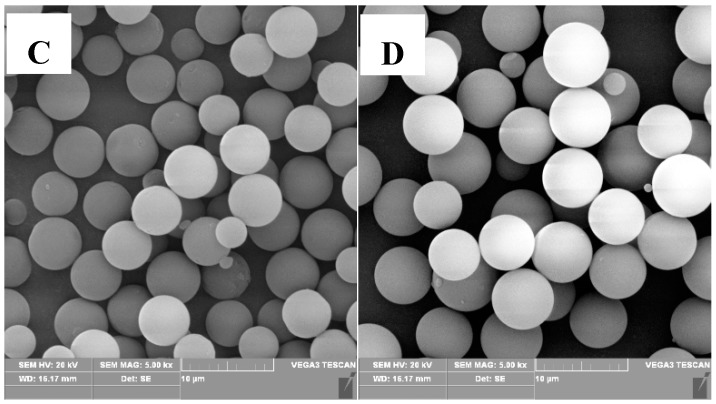
Images of the P(MAA/St) microspheres with different ratios of St:MAA: P-1 (**A**); P-2 (**B**); P-3 (**C**); P-4 (**D**).

Polymer microspheres have carboxyl functional groups, thus they induce the growth of Fe_3_O_4_ on the compound surface. The content of the carboxyl functional groups is controlled by the proportion of MAA monomer in the copolymerization process. Therefore, the influence of St: MAA ratio on the morphology, the particle size distribution and the carboxyl content of P(MAA/St) microspheres were studied. P(MAA/St) microspheres with different St: MAA ratios were prepared. According to the SEM images (Figure 1), all the four kinds of composites had a smooth surface and uniform particle sizes. The particle sizes of the microspheres were about 4–7 μm. When the amount of MAA is too large or too small, small P(MAA/St) microspheres appear in the product. This is because the MAA polymerization activity is higher than St. When a small amount of MAA is used in the system, the amount of copolymer of initial PMAA (polymethylacrylic acid) as the main component is less. This copolymer plays an important role in stabilizing primary nucleating particles. Meanwhile, the content of PMAA in the copolymer affects the solubility of initial P(MAA/St). Premature and homogeneous nucleation led to a wide particle size distribution. Therefore, we can see the small P(MAA/St) microspheres in Figure 1A. An overload of MAA content in the system severely caused a large difference in monomer components in the system before and after the polymerization.

**Figure 2:**
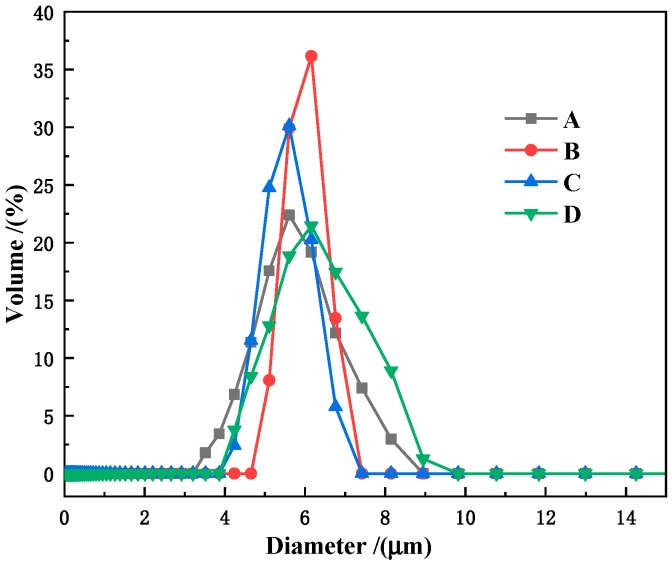
Pore size distribution curves of the four P(MAA/St) microspheres with different ratios of St and MAA: P-1 (**A**); P-2 (**B**); P-3 (**C**); P-4 (**D**).

The particle size distribution of P(MAA/St) microspheres with different St:MAA ratios were calculated and is shown in Figure 2. The particle size distribution of the microspheres all exhibit a monodisperse property. When the MAA content is 0.10 or 0.15 g, the particle size distribution of P(MAA/St) microspheres is the narrowest. The average particle sizes of P-1, P-2, P-3 and P-4 are 5.32, 6.01, 5.45 and 6.55 μm, respectively. The calculated particle size distribution of the microspheres agrees well with the results of SEM observations.

The COOH concentrations on the surface of the P(MAA/St) microspheres were determined by titration, which is shown in Table 2. As the amount of MAA increases, the content of carboxyl groups on the surface of the microspheres increases. The statistical method was used to calculate the surface area of microspheres and the surface density of carboxyl groups on the microspheres, which are shown in Table 2.

### 3.2. Characterization of the P(MAA/St)@Fe_3_O_4_ Magnetic Composite Microsphere

**Figure 3:**
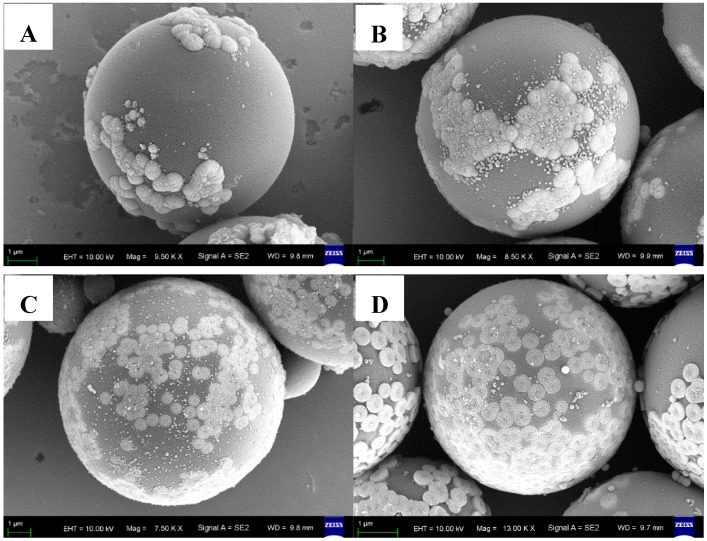
Images of the four P(MAA/St)/Fe_3_O_4_ with different carboxyl content polymer microspheres: MP-1 (**A**); MP-2 (**B**); MP-3 (**C**); MP-4 (**D**).

A series of P(MAA/St)@Fe_3_O_4_ magnetic composite microspheres were prepared by solvothermal processes using P(MAA/St) microspheres with different carboxyl contents on the surface. Magnetic composite microspheres were prepared using P-1, P-2, P-3 and P-4 as the original spheres. They were denoted as MP-1, MP-2, MP-3 and MP-4, respectively. Figure 3 shows SEM images of the obtained P(MAA/St)/Fe_3_O_4_ composite microspheres. The carboxyl content of the P(MAA/St) microspheres is critical for the growth of Fe_3_O_4_ particles on the surface of the microspheres. The Fe_3_O_4_ particles grown on the surface of P(MAA/St) microspheres gradually increases from P1 to P4. Moreover, the area of the Fe_3_O_4_ particles covering the polymer microspheres also increases. However, the Fe_3_O_4_ particle cluster becomes smaller. According to the analysis, as the carboxyl content of the microspheres increases, the chelation of the polymer microspheres with the Fe_3_O_4_ particles is enhanced. Therefore, the growth amount of Fe_3_O_4_ particles increases. Meanwhile, strong chelation increases the growth sites of Fe_3_O_4_ particles. In the case of the same iron source, the Fe_3_O_4_ particle cluster becomes smaller. It can be seen that the thickness of Fe_3_O_4_ layers are different in the SEM images of P(MAA/St)@Fe_3_O_4_ magnetic composite microspheres. With the increasing of the carboxyl groups, the thickness of Fe_3_O_4_ layers decreases.

**Figure 4:**
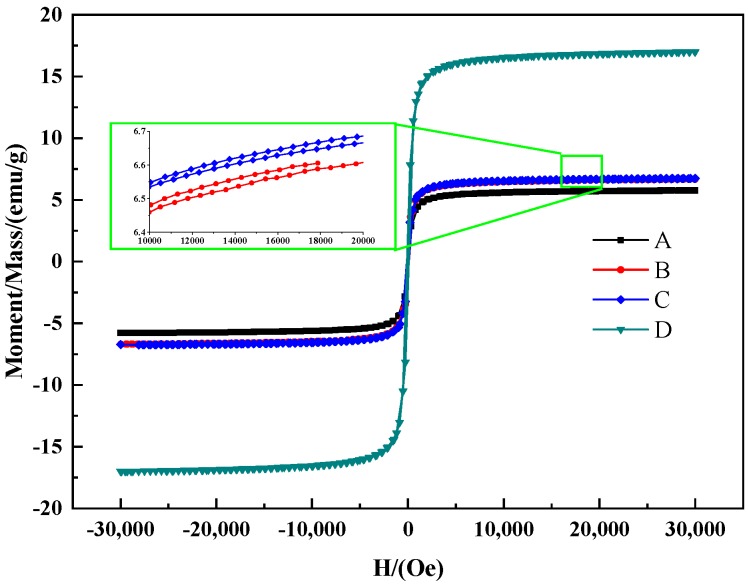
Curves of P(MAA/St)/Fe_3_O_4_ prepared by polymerized microspheres with different carboxyl content on the surface: MP-1 (**A**); MP-2 (**B**); MP-3 (**C**); MP-4 (**D**).

Figure 4 shows the specific saturation magnetization curves of P(MAA/St)/Fe_3_O_4_ prepared from polymer microspheres with different carboxyl content on the surface. It could be seen from the curve that the hysteresis loops of P(MAA/St)/Fe_3_O_4_ magnetic composite microspheres are completely coincidental, proving that these magnetic microspheres were superparamagnetic. The maximum saturation magnetization values of MP-1, MP-2, MP-3 and MP-4 were 5.75, 6.67, 6.73 and 17.00 emu/g. With the increase of the carboxyl content in the composites, the magnetic properties of the composite microspheres increase.

### 3.3. Characterization of the C@Fe_3_O_4_ Magnetic Composite Microsphere

**Figure 5:**
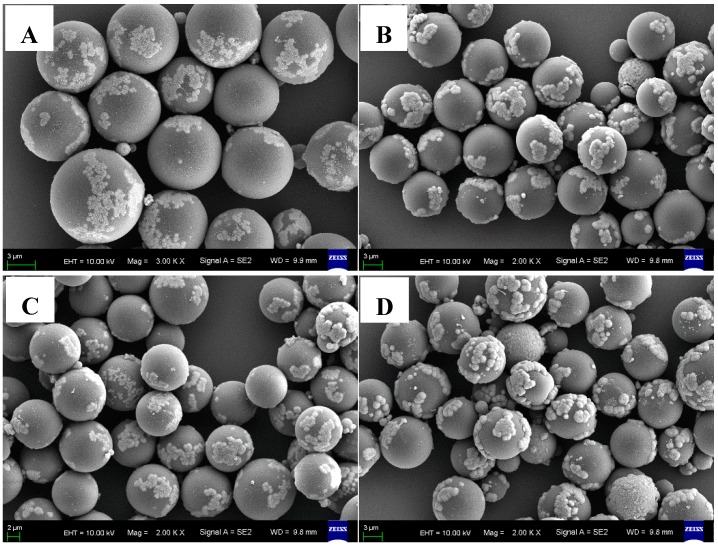
SEM images of the C/Fe_3_O_4_ composite microspheres: MC-1 (**A**); MC-2 (**B**); MC-3 (**C**); MC-4 (**D**).

The MP-1, MP-2, MP-3 and MP-4 were carbonized in a vacuum atmosphere. The obtained samples were named MC-1, MC-2, MC-3 and MC-4, respectively. SEM images are shown in Figure 5, and it can be seen that Fe_3_O_4_ particles on the surface of C@Fe_3_O_4_ magnetic composite microspheres are well maintained after the carbonization process. However, there are some changes in the morphology of the Fe_3_O_4_ nanoparticles and aggregates on the surface of the microspheres. For MP-3 and MP-4, Fe_3_O_4_ nanoparticles are attached to the surface of the polymer microspheres in a toroidal or flat sheet morphology (Figure 3). The morphology of these aggregates changed significantly after carbonization. The Fe_3_O_4_ nanoparticle clusters on the surface of MC-3 and MC-4 microspheres have a higher degree of aggregation, as shown in Figure 5C,D. Simultaneously, the amount of Fe_3_O_4_ on the surface of the microspheres is gradually increased from MC-1 to MC-4. The C@Fe_3_O_4_ magnetic composite microspheres basically maintain the morphology of P(MAA/St)/Fe_3_O_4_ microspheres. The particle size of the microspheres concentrated in 4–6 μm. The reason for the particle size reduction is volume shrinkage due to the loss of organic components during carbonization.

**Figure 6:**
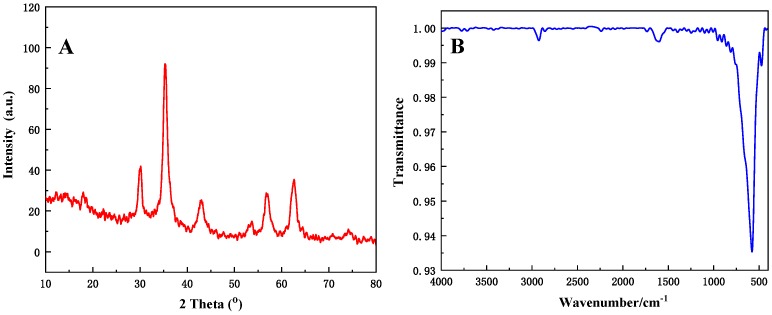
XRD (**A**) and FTIR (**B**) spectra of MC-4.

Due to the consistency of the four kinds of C@Fe_3_O_4_ magnetic composite microspheres in material composition and functional groups, we chose MC-4 composite microspheres as the analyzed objects for material composition characterization. XRD and FTIR analysis of the MC-4 composite microspheres were carried out to determine the composition of the composite microspheres after carbonization. The results were shown in Figure 6. It can be seen from the XRD spectrum of Figure 6A. MC-4 showed obvious crystal diffraction peaks in the range of 20–80°. The diffraction peaks agree well with spinel structure Fe_3_O_4_ (JCPDS 19-0629). XRD peaks at 30.14°, 35.28°, 43°, 56.76° and 62.62° is corresponding to the (220), (311), (400), (511) and (440) planes of Fe_3_O_4_, respectively. Thus, the inorganic component in MC-4 is Fe_3_O_4_. In the FTIR spectrum (Figure 6B), there is no obvious adsorption peak of organic components, indicating that the polymers were completely carbonized. In addition, an obvious absorption peak was found and assigned to Fe-O bonding in MC-4 at 582 cm^−1^. It was confirmed again that the inorganic component was iron oxide.

**Figure 7:**
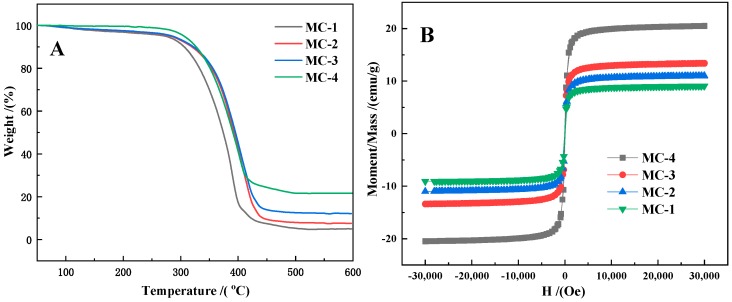
TGA curve (**A**) and VSM (**B**) curve of C/Fe_3_O_4_ composite microspheres.

The different C/Fe_3_O_4_ composite microspheres were characterized by TGA and VSM as shown in Figure 7. It can be seen from the TGA curve that the initial decomposition temperature of C/Fe_3_O_4_ is above 300 °C. According to the TGA results and calculations, the iron oxide contents of samples from MC-1 to MC-4 were calculated to be 4.85%, 7.70%, 12.17% and 21.56%, respectively. According to the VSM curve, carbonization rarely influenced the superparamagnetic of the microsphere material. The maximum saturation magnetization values of MC-1, MC-2, MC-3 and MC-4 were 9.06, 10.97, 13.34 and 20.46 emu/g.

### 3.4. Evaluation of Microwave Absorbing Properties of C@Fe_3_O_4_

**Figure 8:**
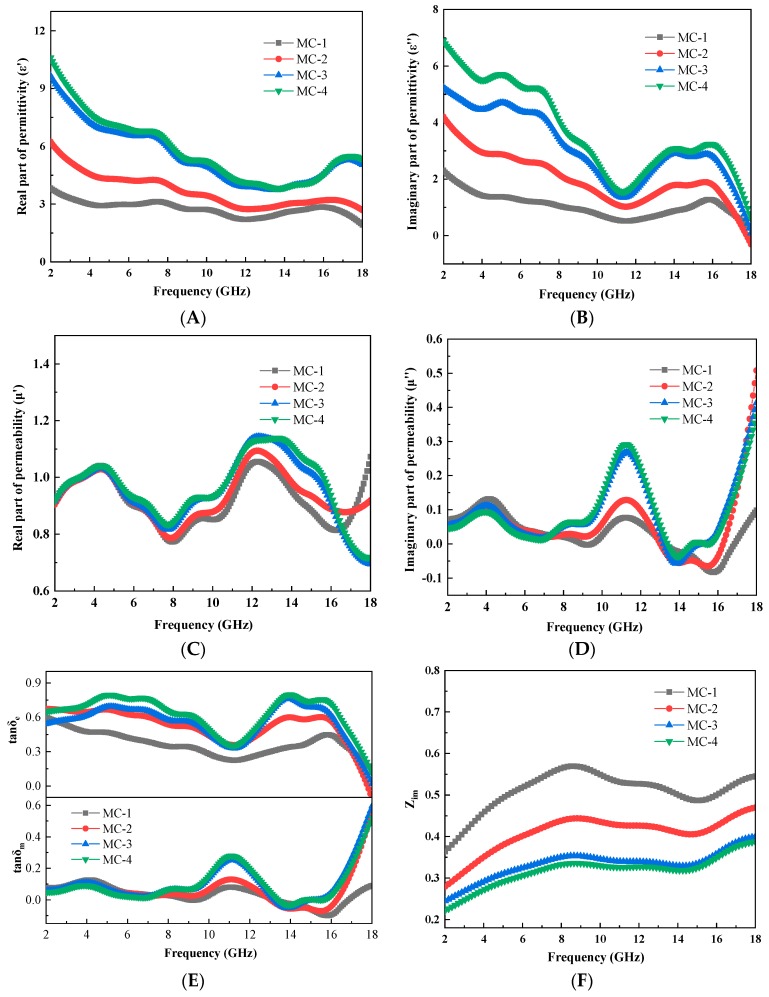
Parameters: the real parts of the complex permittivity for C/Fe_3_O_4_ composite microspheres (**A**); the imaginary parts of the complex permittivity for C/Fe_3_O_4_ composite microspheres (**B**); the real parts of the complex permeability for C/Fe_3_O_4_ (**C**); the imaginary parts of the complex permeability for C/Fe_3_O_4_ (**D**); dielectric loss and magnetic loss curve (**E**); impedance matching curve (**F**).

This section will evaluate the microwave absorbing properties of the obtained C@Fe_3_O_4_. Electromagnetic parameters of the C@Fe_3_O_4_ microspheres were measured by the coaxial method with paraffin as the matrix. The results were shown in Figure 8A–D. The real parts of the complex permittivity (ε′) and complex permeability (μ′) represented the ability of the material to store electric and magnetic field energies, respectively. The imaginary parts of the complex permittivity (ε″) and complex permeability (μ″) represented the ability of the material to lose the energy of electric and magnetic fields, respectively [25,26]. As can be seen from Figure 8A,B, the complex permittivity increased gradually with the increase of the magnetic content of Fe_3_O_4_ on the surface of the carbon sphere. The increasing ε′ indicated a greater ability to store electric fields and more polarization. The larger ε″ indicated a stronger dielectric loss. The imaginary parts of the complex permeability also indicated the same trend. In particular, μ″ turned to negative in the frequency range of 13–17 GHz, which indicated the existence of eddy current loss. This can be attributed to the eddy current, which produced a reverse magnetic field. It counteracted the intrinsic magnetic field, causing the magnetic permeability to become negative (Figure 8D). The dielectric loss tangent (tan δ_e_ = ε″/ε′) and the magnetic loss tangent (tan δ _m_ = μ″/μ′) were used to indicate the dielectric loss and the magnetic loss [27]. The relationship curves of the dielectric loss and magnetic loss versus frequency were shown in Figure 8E. It was seen that the dielectric loss was significantly larger than the magnetic loss. This indicated that the dielectric loss was dominant. The two curves were symmetrically up and down, demonstrating the complementarity between dielectric loss and magnetic loss. Moreover, the dielectric loss and magnetic loss both increased with the increase of Fe_3_O_4_ loading. The reason was described as that the magnetic properties of the composite absorbing materials increased with the increase of Fe_3_O_4_ loading. Therefore, the magnetic loss performance was further improved. At the same time, the interface between the carbon spheres and the Fe_3_O_4_ particles also increased. This resulted in more interfacial polarization and improved the dielectric loss capability. The comprehensive absorbing properties of the absorbing materials were also affected by the impedance matching characteristics. The impedance matching curves of C@Fe_3_O_4_ magnetic composite microspheres with different magnetic contents were shown in Figure 8F. It was clear to see that the characteristic impedance ($Z_{im}=\sqrt{\mu_{0}/\varepsilon_{0}}\sqrt{\mu_{r}/\varepsilon_{r}}$) of the material decreased continuously with the increase of Fe_3_O_4_ particle loading. The outcomes illustrated that the impedance matching had reduced. This will have a negative impact on the overall absorbing properties of C@Fe_3_O_4_ magnetic composite microspheres.

The electromagnetic wave absorption efficiency of the absorbing materials was expressed by the reflection loss (RL). According to the transmission line theory, the calculation formula was as follows [28]:

(1) $$RL=20\;\mathrm{lg}\left|\frac{Z_{in}-1}{Z_{in}+1}\right|,$$

(2) $$Z_{in}=\sqrt{\frac{\mu_{r}}{\varepsilon_{r}}}\tan h\left[j\left(\frac{2\pi fd}{c}\right)\sqrt{\mu_{r}\varepsilon_{r}}\right],$$

where *μ_r_* and *ε_r_* were the complex permeability and complex permittivity of the materials, *f* was the frequency of the electromagnetic wave, *d* was the thickness of the absorbing materials, and *c* was the velocity at which the electromagnetic wave propagated in the vacuum.

**Figure 9:**
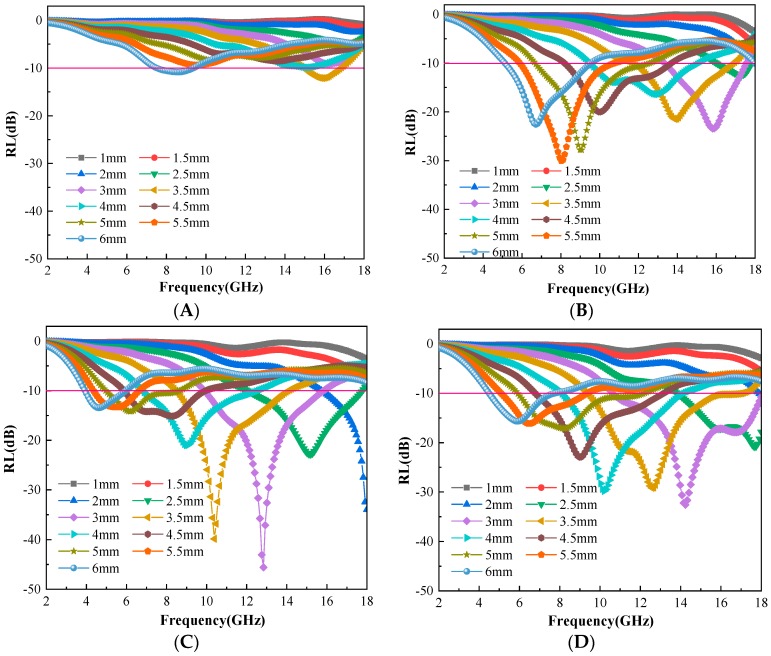
Loss of C/Fe_3_O_4_: MC-1 (**A**); MC-2 (**B**); MC-3 (**C**); MC-4 (**D**).

Figure 9 gives the reflection loss curves of C@Fe_3_O_4_ magnetic composite microspheres with different magnetic contents at different matching thicknesses. It can be seen that the reflection loss first increased and then decreased with the increase of magnetic contents. The order of the reflection loss was −12.1 dB, −30.1 dB, −45.6 dB and −32.6 dB. The corresponding matching frequencies were 15.9 GHz, 8.0 GHz, 12.8 GHz and 14.2 GHz, respectively. The matching thickness was 3.5 mm, 5.5 mm, 3 mm and 3 mm. In particular, MC-3 had the best absorbing properties. Its effective bandwidth with an absorption rate greater than 90% (RL < −10 dB) reached 5.9 GHz. The above results show that the absorbing properties of C@Fe_3_O_4_ magnetic composite microspheres increased first and then decreased with the increase of magnetic contents. This is because the dielectric loss and magnetic loss of the C@Fe_3_O_4_ both increased with the increase of the magnetic contents while the impedance matching decreased. Therefore, the ultimate electromagnetic wave loss capability was controlled by these two factors. The optimum magnetic content of the C@Fe_3_O_4_ was 12.17%.

**Figure 10:**
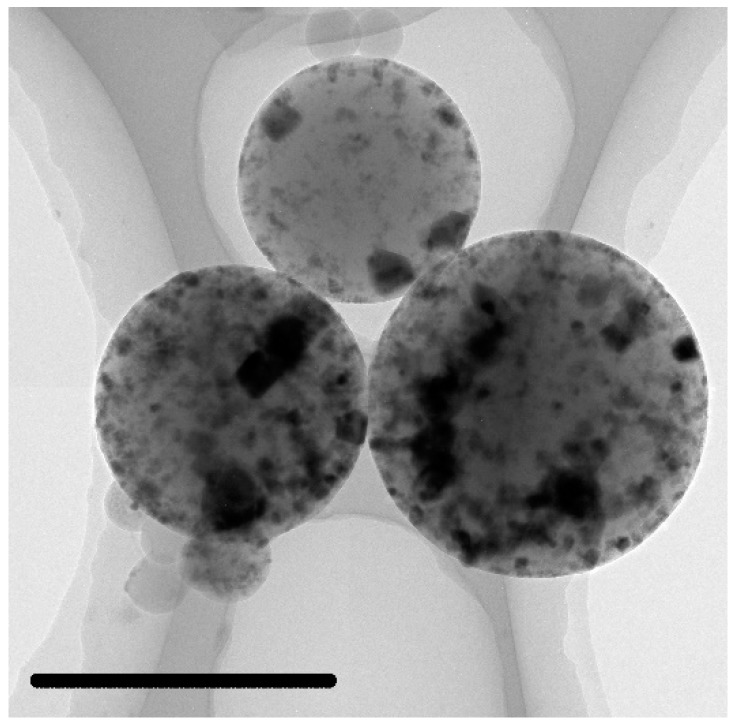
Image of C@Fe_3_O_4_ (MC-3), the scale bar was 5 μm.

In order to further reveal the electromagnetic wave loss mechanism of C@Fe_3_O_4_, the electromagnetic parameters of MC-3 were analyzed in depth. The reason was its excellent microwave absorbing properties. The TEM image of MC-3 is shown in Figure 10. Inorganic nanoparticles were the materials with high mass thickness contrast. Their distribution was partially clustered. The space in the microspheres with low mass thickness contrast was carbon.

**Figure 11:**
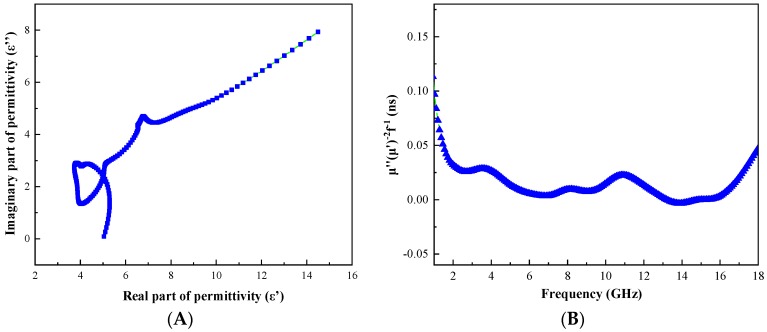
Plots of ε″ versus ε′ (**A**) and μ″(μ)′^−2^f^−1^ versus frequency (**B**) of dielectric constant.

According to Debye theory, the complex permittivity can derive the following relationship [29,30]:

(3) $${\left(\varepsilon^{\prime }-\frac{\varepsilon_{s}+\varepsilon_{\infty }}{2}\right)}^{2}+{\left(\varepsilon^{\prime \prime }\right)}^{2}={\left(\frac{\varepsilon_{0}-\varepsilon_{\infty }}{2}\right)}^{2},$$

where *ε_s_* is the static dielectric constant, *ε_∞_* is the limit of the relative dielectric constant at high frequencies, and *ε*_0_ is a dielectric constant in vacuum. From this expression, it is known that the curve about ε′ and ε″ is a semicircle, which is called the Cole-Cole semicircle. Each semicircle corresponded to a Debye relaxation process [3]. A plot of ε″ versus ε′ is presented in Figure 11A. A clear Cole-Cole semicircle can be seen from the figure. This confirmed the existence of the Debye relaxation process. The eddy current loss effect was judged by the equation $C_{0}=\mu \prime \prime {\left(\mu \prime \right)}^{-2}f^{-1}=2\pi \mu_{0}d^{2}\delta$. If the eddy current loss existed in the electromagnetic wave absorption process, *C*_0_ was always constant as the frequency changed [31,32]. The relationship between *C*_0_ and the frequency of C@Fe_3_O_4_ is shown in Figure 10B. The curve was approximately straight in the range of 6–9 GHz and 14–16 GHz. This further indicated the presence of eddy current losses in this frequency range. The natural resonance effect was described by the following formula [11]:

(4) $$2\pi f_{r}=rH_{\partial },$$

(5) $$H_{\partial }=\frac{4\left|K_{1}\right|}{3\mu_{0}M_{S}},$$

(6) $$\frac{1}{2}\mu_{0}M_{S}H_{C},$$

where *μ_o_* was the vacuum permeability (4π × 10^−7^ H/m), *r* was the gyromagnetic ratio, *H_α_* was the anisotropic energy, *K*_1_ was the anisotropy coefficient, and *M_s_* and *H_c_* were the saturation magnetization and coercive force, respectively. It was inferred that the resonant frequency depended on the effective anisotropy field. The effective anisotropy field was related to the material coercivity value [33]. Combined with the hysteresis loop (the inset of Figure 7B), it can be seen that the C@Fe_3_O_4_ had a larger coercive force. The higher the coercivity is, the higher frequency resonance will be generated. This will improve the magnetic loss performance of the material effectively [34].

## 4. Conclusions

In this study, C@Fe_3_O_4_ magnetic microsphere absorbing materials with controllable Fe_3_O_4_ contents were successfully prepared. The magnetic contents of the surface of C@Fe_3_O_4_ were controlled by adjusting the carboxyl contents of the surface of the polymer precursor microspheres. The absorbing performance analysis showed that the magnetic contents had a significant effect on the absorbing properties of C@Fe_3_O_4_. With the increase of magnetic contents, the dielectric loss and magnetic loss increased, and the characteristic impedance decreased. Benefiting from the effective compounding of carbon and Fe_3_O_4_, C@Fe_3_O_4_ had multiple loss mechanisms, including interfacial polarization, Debye relaxation, eddy current loss, and natural resonance. The C@Fe_3_O_4_ magnetic microspheres exhibited excellent absorption properties. The maximum reflection loss at 12.8 GHz reached −45.6 dB when the matching thickness was 3 mm. Moreover, the effective bandwidth of RL < −10 dB was 5.9 GHz, which demonstrates that the C@Fe_3_O_4_ magnetic microspheres are promising materials for microwave absorption applications.

## Figures and Tables

**Table 1:** Effect of monomer ratio on the performance of P(MAA/St) microspheres.

Sample No.	P-1	P-2	P-3	P-4
MAA (g)	0.05	0.10	0.15	0.20
St (g)	6.30	6.25	6.20	6.15

**Table 2:** Carboxyl concentration of P(MAA/St) microspheres prepared with different St:MAA ratios.

Sample No.	P-1	P-2	P-3	P-4
Carboxyl content (mmol/g)	0.07	0.15	0.23	0.40
Surface density of carboxyl groups (μmol/m^2^)	23.14	88.03	92.64	142.80
